# Cell surface expression of nucleolin mediates the antiangiogenic and antitumor activities of kallistatin

**DOI:** 10.18632/oncotarget.23346

**Published:** 2017-12-16

**Authors:** Xiao-Ping Huang, Xiao Wang, Xiao-Lan Xie, Gao-Ping Zhang, Feng-Jiao Lv, Wen-Ting Weng, Fei Qiu, Zhao-Fa Li, Jun-Sheng Lin, Yong Diao

**Affiliations:** ^1^ College of Chemical Engineering and Materials Sciences, Quanzhou Normal University, Quanzhou, China; ^2^ Institute of Molecular Medicine, Huaqiao University, Quanzhou, China

**Keywords:** kallistatin, nucleolin, endothelial cell, tumor angiogenesis

## Abstract

Kallistatin is a unique serine proteinase inhibitor and heparin-binding protein. A previous study conducted by our group indicated that kallistatin has antiangiogenic and antitumoral activities. In the present study, we report that kallistatin specifically binds to membrane surface-expressed nucleolin with high affinity. Antibody-mediated neutralization or siRNA-induced nucleolin knockdown results in loss of kallistatin suppression of endothelial cell proliferation and migration *in vitro* and tumor angiogenesis and growth *in vivo*. In addition, we show that kallistatin is internalized and transported into cell nuclei of endothelial cells via nucleolin. Within the nucleus, kallistatin inhibits the phosphorylation of nucleolin, which is a critical step required for cell proliferation. Thus, we demonstrate that nucleolin is a novel functional receptor of kallistatin that mediates its antiangiogenic and antitumor activities. These findings provide mechanistic insights into the inhibitory effects of kallistatin on endothelial cell growth, tumor cell proliferation, and tumor-related angiogenesis.

## INTRODUCTION

Angiogenesis denotes the proliferation of endothelial cells and outgrowth of new capillaries from preexisting vessels. This process is critical for tumor growth and facilitates tumor invasion and metastasis. Angiogenic vessels possess unique morphological and molecular properties that distinguish them from normal vessels. Among molecular markers of neovessels are endothelial growth factor receptors, vascular growth factor receptors, specific integrins, nucleolin, some proteolytic enzymes and extracellular matrix proteins that allow the generation of a permissive niche for new vascular growth [1–3], as well as membrane proteins of unknown function [4, 5]. Therefore, the use of antiangiogenic molecules has been widely adopted as therapeutic modality for cancer treatment. Kallistatin is a heparin-binding protein and serine proteinase inhibitor (serpin) that was first isolated from plasma and showed to act as a tissue kallikrein inhibitor [6, 7]. It was later shown to exert a variety of biological effects in response to pathological conditions such as abnormal blood pressure regulation, tumor formation, and inflammation [6, 8, 9]. Previous studies conducted by our research group demonstrated that adenovirus-mediated kallistatin gene delivery inhibited human colorectal cancer, hepatocellular carcinomas, and small cell lung cancer mainly through inhibition of angiogenesis [10–12]. The anti-angiogenic functions of kallistatin are associated with its structure. For example, the heparin-binding domain is essential in suppressing VEGF-mediated endothelial cell proliferation and migration [13]. In many ligand-receptor systems initial ligand binding occurs onto abundantly expressed low-affinity receptors; these in turn recruit the ligand to the cell surface, where it activates high-affinity receptors that transduce the corresponding signals. Heparan sulfate proteoglycans (HSPGs) are the most ubiquitous low-affinity receptors shaping cellular responses to numerous growth factors. Importantly, HSPGs interact with integrins at the cell surface to regulate cell adhesion and motility [14]. Kallistatin acts by competing with VEGF and bFGF for binding to HSPGs. This occurs at a low affinity-binding site, and suppresses pro-angiogenic signaling by these two growth factors [6].

Although several kallistatin-binding proteins such as integrin β3, Krüppel-like factor 4 (KLF4), and lipoprotein receptor-related protein 6 have been reported as kallistatin receptors [12, 15, 16], the exact mechanisms underlying kallistatin effects remain unclear. Moreover, the involvement of these putative kallistatin receptors in the antitumoral action of kallistatin remains elusive. The biomarkers present on the tumor vasculature perform specific biological functions related to the onset and maintenance of the neoangiogenic process. Nucleolin was initially reported as a surface expression marker in hepatocarcinoma cells, where it served as a low affinity receptor for diverse ligands including some growth factors [17]. A previous study has shown that enhanced expression of nucleolin on the surface of both tumor and tumor-associated endothelial cells was correlated with the development of tumor vasculature [18, 19]. In endothelial cells, nucleolin expression is stimulated by VEGF, and functional inhibition and/or downregulation of nucleolin expression on the surface of endothelial cells has been shown to block migration and inhibit capillary-tubule formation [20, 21].

We previously reported the binding of kallistatin to integrin β3 on the surface of human lung cancer NCI-H446 cells, an interaction that negatively affected tumor cell proliferation, migration, and survival [12]. Shen *et al*. identified KLF4, a prominent transcription factor possessing strong nuclear localization sequences, as another binding partner of kallistatin that purportedly mediates its nuclear translocation [16]. This interaction revealed a novel anti-inflammatory effect of kallistatin, mediated by increased endothelial nitric oxide synthase (eNOS) expression and NO production in endothelial cells.

**Figure 1:**
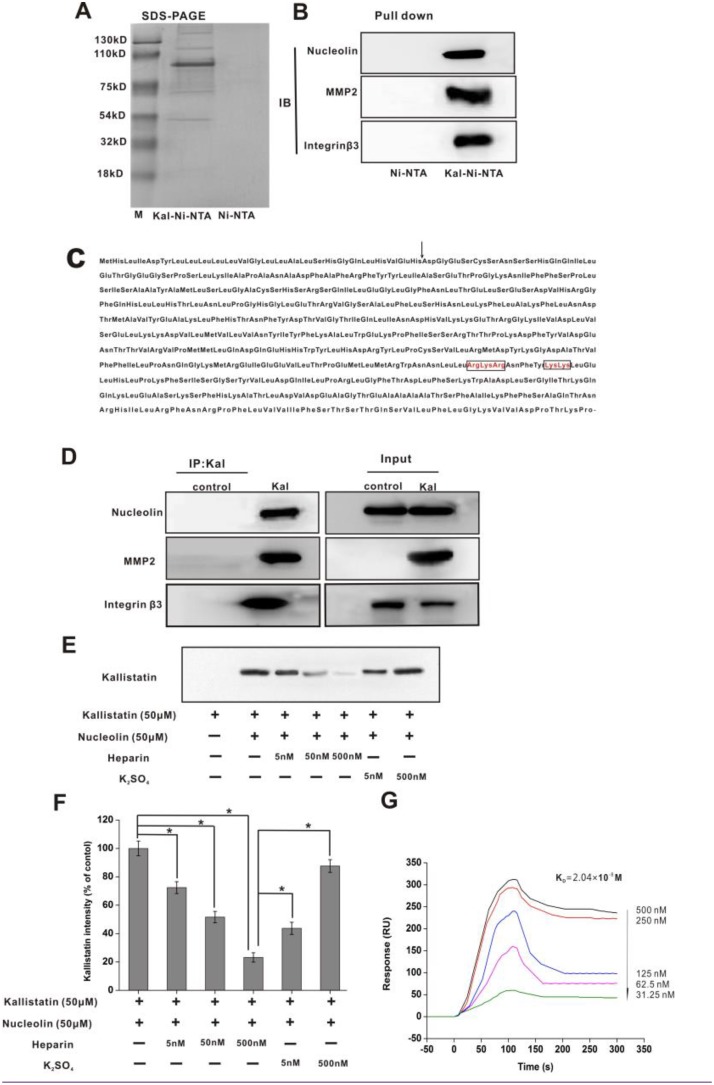
Nucleolin, integrin β3, and MMP2 are kallistatin-binding partners (**A**) SDS-PAGE (Coomassie blue staining) was performed on the eluted fraction of Ni-NTA and Kal-Ni-NTA affinity chromatography. (**B**) IB results of pull-down samples from Ni-NTA and Kal-Ni-NTA affinity chromatography. (**C**) Amino acid sequence of kallistatin. The arrow indicates the potential cleavage site of the signal peptide. The basic residues in the putative heparin-binding regions are in boxes. (**D**) Immunoprecipitation and IB of kallistatin-binding proteins using anti-kallistatin and control anti-rabbit IgG antibodies. (**E**) IB images of kallistatin-nucleolin complex precipitated by an anti-nucleolin antibody and detected using an anti-kallistatin antibody. (**F**) Semi-quantification of kallistatin expression. Data are expressed as mean ± SD from three independent experiments (*N* = 3). ^*^P < 0.05. (**G**) Real-time surface plasmon resonance (SPR) kinetic binding sensorgrams depicting the interaction of kallistatin with nucleolin at the indicated concentrations. The *K*d that was used as an index of the binding interaction of kallistatin with nucleolin was estimated to be 2.04 × 10^−8^ M from these curves.

However, at present the list of kallistatin receptors appears to be inconclusive. Nucleolin acts as a functional receptor for endostatin, a type XVIII collagen-derived protein with potent anti-angiogenic and anticancer effects. The interaction of endostatin with nucleolin, and its internalization, is facilitated by arginine clusters in endostatin's heparin-binding motif [22]. Because the structures of kallistatin and endostatin are very similar, i.e. they both contain a heparin-binding site (Figure 1C), we hypothesized that nucleolin may also mediate the antiangiogenic actions of kallistatin. In the present study, kallistatin expression, the mechanisms underlying its antiangiogenic activity, and the role played by nucleolin were assessed in cultured human umbilical vein endothelial cells (HUVECs) and in tumor-bearing mice. Our results show that nucleolin and kallistatin are overexpressed in tumor-associated blood vessels, and define a role for nucleolin as a kallistatin receptor responsible for its suppressive actions on tumor angiogenesis and cancer cell growth.

## RESULTS

### Nucleolin is a kallistatin-binding protein

HUVECs were selected to identify kallistatin-binding partner proteins due to the sensitivity of these cells to kallistatin treatment, as evidenced by endothelial cell proliferation and migration assays [6, 23] which suggested that HUVECs express functional kallistatin receptor(s). Pull-down assays revealed 5 proteins eluted in the Kal-Ni-NTA affinity column, which had apparent molecular weights of 200, 110, 75, 90, and 50 kDa as demonstrated by SDS-PAGE under reducing conditions (Figure 1A). Subsequently, these proteins were identified as nucleolin (110 kDa), MMP2 (49 KDa), and integrin β3 (200 kDa) by peptide mass fingerprinting using MALDI-TOF MS. Immunoblot (IB) analysis was further applied to validate the identities of these proteins using specific monoclonal antibodies (Figure 1B).

To validate these findings, HUVECs were treated with kallistatin for 4 h. Following treatment, immunoprecipitation of the cell lysates was carried out with an antibody raised against kallistatin, following by separation of complexes by SDS-PAGE and further immunoblotting. These analyses confirmed that kallistatin co-precipitated with nucleolin, integrin β3 and MMP2 (Figure 1D). Soluble heparin was used for coimmunoprecipitation (co-IP) experiments to assess whether kallistatin interacts with nucleolin via its heparin-binding sites. The results indicated that increasing concentrations of soluble heparin induced gradual disassociation of the nucleolin and kallistatin complex. In contrast, a 1,000-fold excess concentration of sodium sulfate was insufficient to disrupt this interaction (Figure 1E, 1F). These data suggested that kallistatin interacts with nucleolin via multiple heparin-binding sites. Next, we used real-time surface plasmon resonance (SPR) to determine the binding affinity between kallistatin and nucleolin. A high binding affinity, represented by an estimated equilibrium dissociation constant (*K*d) of 2.04 × 10^–8^ M, was estimated for this interaction (Figure 1G).

### Cell-surface distribution of nucleolin

**Figure 2:**
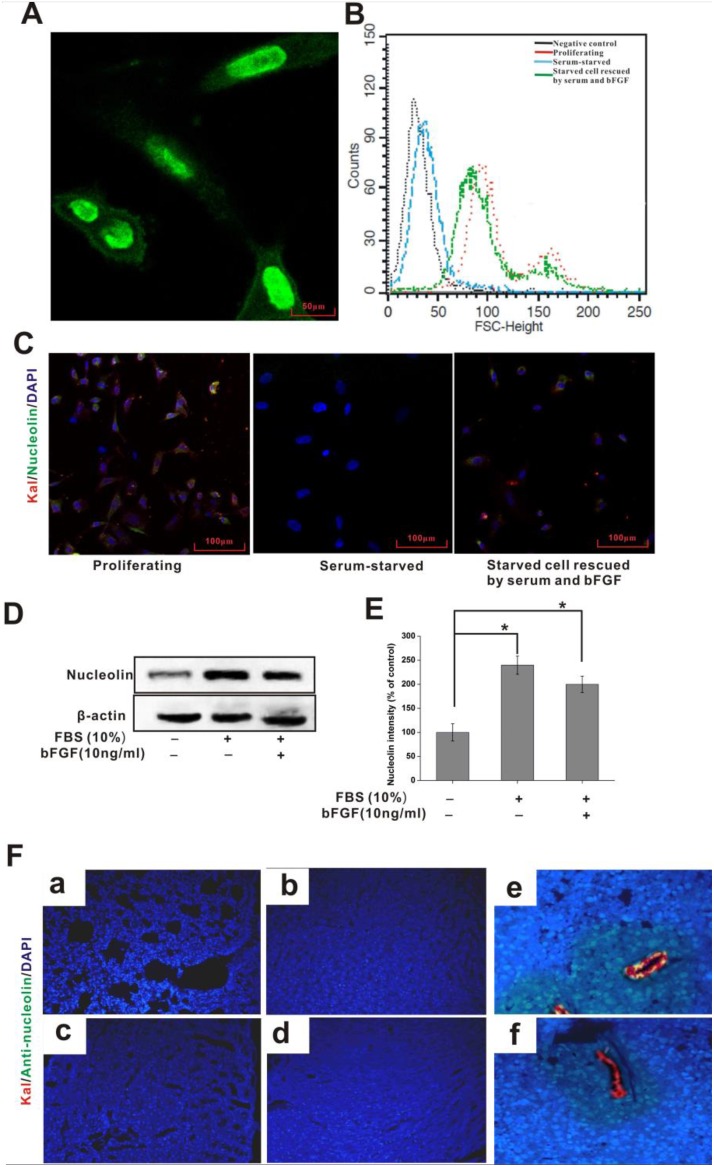
Cellular distribution of nucleolin and kallistatin *in vitro* and *in vivo* (**A**) Immunofluorescence staining of nucleolin in cultured HUVECS. (**B**) FACS analysis of surface-bound nucleolin in HUVECs grown under different conditions. (**C**) Indirect immunofluorescence colocalization of nucleolin (green) and kallistatin (red) on HUVECs under different growth conditions. DAPI (blue) was used to stain cell nuclei. (**D**) IB analysis of cell surface kallistatin in HUVECs grown in different conditions. (**E**) Semi-quantification of nucleolin relative expression. Data are expressed as mean ± SD from three independent experiments (*N* = 3). ^*^P < 0.05. (**F**) Kallistatin plus either anti-nucleolin or nonimmune rabbit IgG (control) antibody were simultaneously injected intravenous into tumor-bearing mice After 1 h, the distribution of kallistatin (red) and anti-nucleolin antibody (green) in lung (a), liver (b), kidney (c), heart (d), tumor (simultaneously injected kallistatin and anti-nucleolin antibody ) (e), and control tumor (simultaneously injected kallistatin and nonimmune rabbit IgG) (f) were detected with Alexa Fluor 555-conjugated goat anti-rabbit and Alexa Fluor 488-conjugated goat anti-mouse IgG, respectively. DAPI (blue) indicates cell nuclei in the field.

Localization to the cell surface is required for nucleolin to serve as a kallistatin receptor. Nucleolin is primarily localized in both the nucleus and cytoplasm, although localization to the a cell surface has been also shown to occur [24, 25]. Immunofluorescence microscopy indicated that nucleolin was predominantly expressed in the nucleus, with minor expression seen at the cell membrane (Figure 2A). Since previous evidence indicated that nucleolin distribution on the cell surface changes with cell growth stages, we used flow cytometry to analyze nucleolin expression patterns in HUVECs under three different growth conditions, namely in serum-supplemented medium, during serum starvation, and afterwards, during rescuing from serum starvation in the presence of both serum and bFGF. The results indicated positive association of membrane-surface expression of nucleolin with *in vitro* cell growth conditions (Figure 2B). Surface levels of kallistatin and nucleolin were subsequently investigated using confocal microscopy on HUVECs grown in the same conditions. Results showed that nucleolin and kallistatin levels decreased in parallel during 24 h culture in serum-free medium. Following serum restitution and bFGF addition for 12 h, partial recovery of cell-surface nucleolin expression was observed (Figure 2C). Furthermore, formation of kallistatin-nucleolin complexes was detected on proliferating HUVECs (Figure 2D, 2E); complex formation was reduced during serum-starvation, and increased instead following serum and bFGF addition.

Next, the interaction between kallistatin and nucleolin was assessed in the vasculature of tumor-bearing mice after *in vivo* injection of corresponding antibodies. Interestingly, no lung, hepatic, renal or cardiac signal was observed for both kallistatin and nucleolin antibodies (Figure 2F). Instead, a clear signal was detected on the surface of tumor-associated blood vessels (Figure 2F). Control experiments using intravenous injection of nonimmune IgG and kallistatin showed no IgG binding to either normal or tumor tissues. These results indicate selective colocalization of kallistatin and nucleolin on the surface of tumor-associated blood vessels, rather than in normal blood vessels and healthy organs and tissues.

### Nucleolin mediates kallistatin internalization in cultured HUVECs

**Figure 3:**
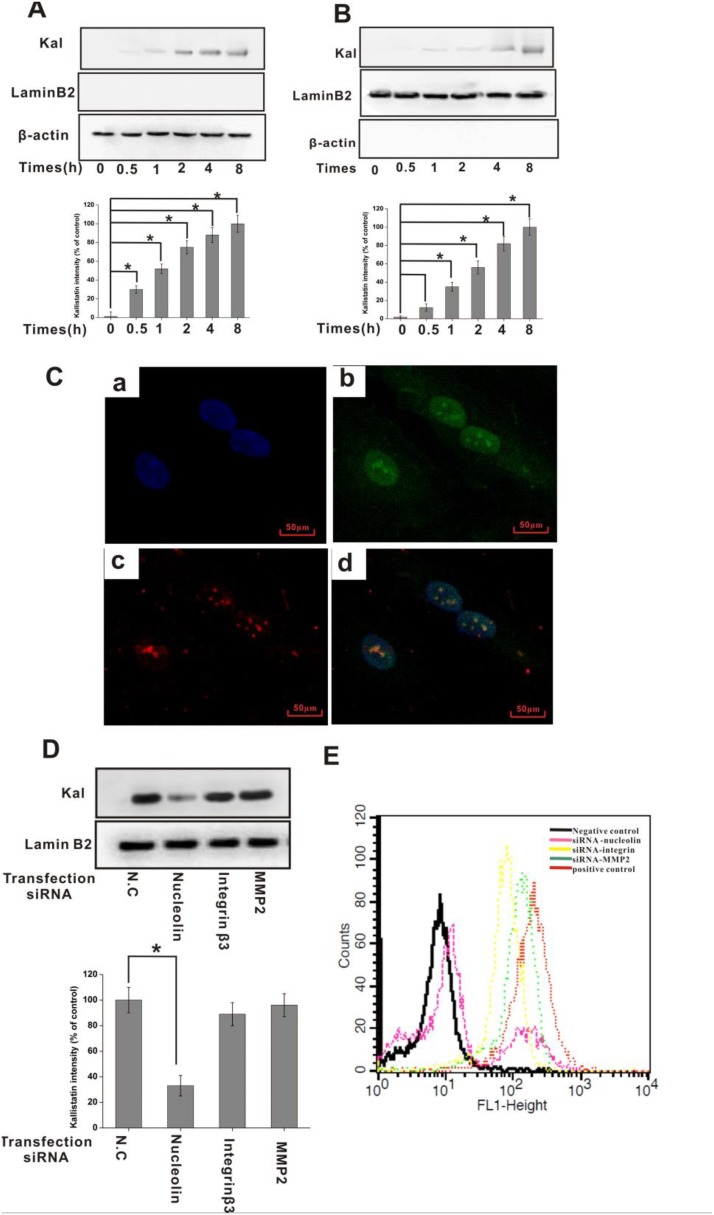
Nucleolin mediates kallistatin entry into endothelial cells HUVECs were incubated with kallistatin for the indicated times and homogenized to obtain cytosolic and nuclear cell fractions. Kallistatin expression in the cytosol (**A**) and nucleus (**B**) was detected by IB with an anti-kallistatin antibody and semi-quantified (histograms). Lamin B2 and GAPDH served as controls for nuclear and cytosolic fractions, respectively. Data are expressed as mean ± SD from three independent experiments (*N* = 4). ^*^P < 0.05. (**C**) HUVECs bind and internalize kallistatin. DAPI (blue) indicates cell nuclei in the field. Nucleolin is stained green; Kallistatin is stained red. (**D**) Following transfection with siRNA for 48 h, HUVECs were incubated with kallistatin for 2 h. Nuclear localization of kallistatin in siRNA-transfected HUVECs was detected by IB with an anti-kallistatin antibody and semi-quantified (histogram). Lamin B2 served as a loading control. Data are expressed as mean ± SD from three independent experiments (*N* = 4). ^*^P < 0.05. (**E**) Flow cytometric analysis of cell surface's kallistatin in siRNA-transfected HUVECs. Scrambled siRNA-transfected HUVECs labeled with Alexa Fluor 647-labeled goat IgG plus or minus anti-kallistatin antibody served as positive and negative controls, respectively.

Nucleolin has been shown to traffic between the cell surface, cytoplasm, and nucleus, and to mediate internalization and nuclear translocation of certain cytokines [26–28]. Since cell internalization is an essential step for the manifestation of kallistatin bioactivity, we investigated the dynamics of kallistatin internalization and its subcellular distribution in HUVECs incubated with kallistatin for various time periods. Following extraction of nuclear and cytosolic protein fractions, kallistatin detection was performed using immunoblotting, while immunofluorescence was used in intact cells. Results indicated that cytosolic kallistatin levels remained relatively constant from 0.5 to 8 h (Figure 3A), whereas nuclear localization increased gradually from 1 to 8 h (Figure 3B, 3C). To explore the role of nucleolin in the internalization and subsequent nuclear translocation of kallistatin, expression analyses were performed in HUVECs after siRNA-mediated nucleolin knockdown. Both immunoblotting (Figure 3D) and flow cytometry (Figure 3E) indicated that nuclear and surface kallistatin levels were significantly decreased when nucleolin was knocked down. In contrast, surface binding and translocation of kallistatin to the nucleus were not significantly altered upon knockdown of MMP2 and integrin β3 (Figure 3D, 3E). However, since nucleolin knockdown did not fully prevent kallistatin binding to HUVECs, it is highly likely that other molecules function as kallistatin binding partners or receptors in these cells.

### Nucleolin mediates the inhibitory effect of kallistatin on cell viability

**Figure 4:**
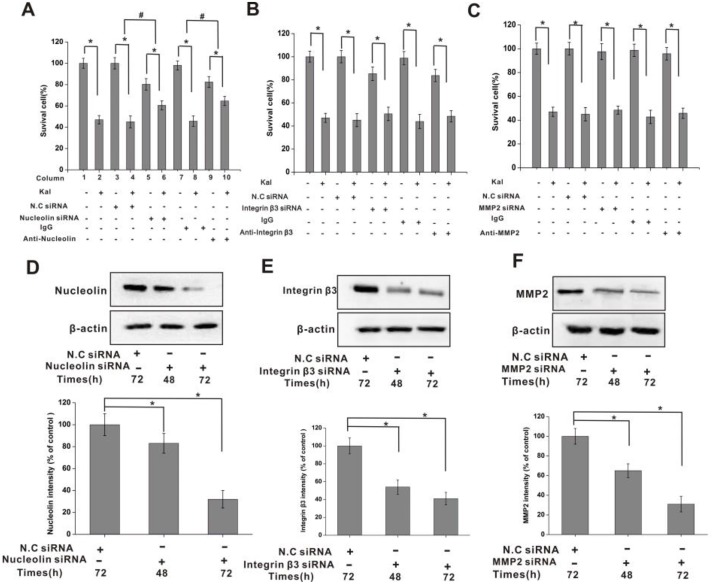
Nucleolin mediates the inhibitory effects of kallistatin on HUVECs viability (**A**) Summary histogram showing the inhibitory effect of kallistatin on the viability of HUVECs. Kallistatin-mediated viability inhibition, measured by the MTT assay, was attenuated by pre-treatment with nucleolin-targeted siRNA or anti-nucleolin antibody. N.C. siRNA and IgG were used as negative controls. *N* = 6×3 for each group. ^*^P < 0.05, ^#^*P* < 0.05 for columns 3 vs 4 and 5 vs 6. (**B**) The inhibitory effect of kallistatin on the viability of HUVECs was not affected by pre-treatment with integrin β3 siRNA or anti-integrin antibody. N.C siRNA and IgG were used as negative controls. *N* = 6×3 for each group. ^*^P < 0.05. (**C**) The inhibitory effect of kallistatin on the viability of HUVECs was not affected by pre-treatment with MMP2 siRNA or anti-MMP2 antibody. N.C siRNA and IgG were used as negative controls. *N* = 6 × 3 for each group. ^*^P < 0.05. Time-dependent, siRNA-mediated downregulation of nucleolin (**D**), integrin β3 (**E**) and MMP2 (**F**) expression in HUVEC cells. Semi-quantification data is shown on respective histograms. The housekeeping protein β-actin was used as loading control for protein expression normalization. Data are expressed as mean ± SD from three independent experiments (*N* = 4). ^*^P < 0.05.

To investigate whether specific surface expression of nucleolin on proliferating endothelial cells is required for kallistatin antiangiogenic activities, cell viability assays were conducted in HUVECs with intact or silenced nucleolin, integrin β3, and MMP2. Incubation with kallistatin decreased cell viability in HUVECs, and this effect was attenuated by siRNA-mediated nucleolin knockdown (Figure 4A), but not by integrin β3 and MMP2 silencing (Figure 4B, 4C). In addition, an anti-nucleolin antibody, but not a nonimmune IgG antibody, was also able to attenuate the decrease in cell viability induced by kallistatin (Figure 4A). Silencing efficacy of the siRNAs was evaluated by western blotting. Nucleolin expression inhibition was approximately 80% (Figure 4D), while a slightly lower knockdown efficacy was achieved for integrin β3 (Figure 4E) and MMP2 (Figure 4F).

### Nucleolin knockdown attenuates kallistatin-mediated inhibition of cell proliferation and migration

**Figure 5:**
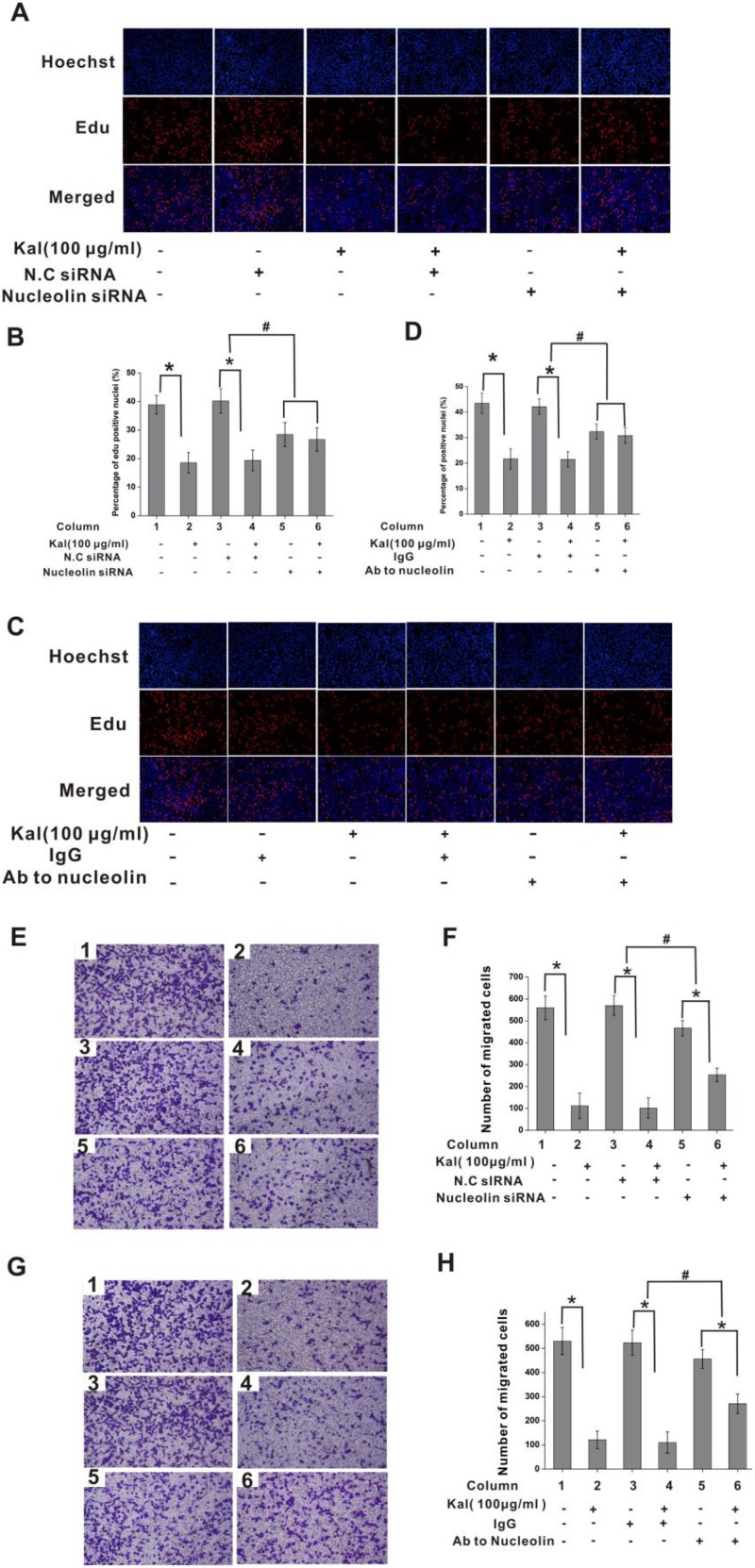
Nucleolin mediates the antiproliferative effect of kallistatin on endothelial cells (**A**) Representative images of EdU cell proliferation assay. HUVECs were transfected with N.C. siRNA or nucleolin-targeted siRNA and exposed to kallistatin (100 μg/mL) or left untreated. (**B**) Quantification of EdU-positive HUVEC cells from pictures like those shown in A. Data are presented as mean ± SD (*N* = 6). ^*^P < 0.05. ^#^*P* < 0.05 for columns 3 vs 4 and 5 vs 6. (**C**) Representative images of EdU-stained HUVECs pre-treated with nonimmune IgG (10 μg/mL) or anti-nucleolin antibody (10 μg/mL) and exposed to kallistatin (100 μg/mL) or left untreated. (**D**) Quantification of EdU-positive cells from pictures like those shown in C. Data are presented as mean ± SD (*N* = 6). ^*^P < 0.05. ^#^*P* < 0.05 for columns 3 vs 4 and 5 vs 6. (**E**, **G**) Representative images of cell migration experiments. HUVECs were transfected with saline, NC siRNA, or nucleolin-targeted siRNA (E) or pre-treated with saline (control), nonimmune IgG (10 μg/mL), or anti-nucleolin antibody (G), and exposed to kallistatin (100 μg/mL) or left untreated before being subjected to transwell migration assays (*N* = 5). (**F**, G) Quantification of cell migration from experiments shown in E (F) and G (**H**). Data are presented as mean ± SD (*N* = 6). ^*^P < 0.05; ^#^*P* < 0.05 for columns 3 vs 4 and 5 vs 6 .

Consequently, the impact of kallistatin on HUVEC proliferation was assessed by labeling cell nuclei with 5-ethynyl-2´-deoxyuridine (EdU), a thymidine analogue that allows measurement of total proliferating cell numbers in cultures. Results showed significant proliferation inhibition in kallistatin-treated HUVEC cultures; this effect was attenuated by nucleolin-knockdown, but not by scrambled si-RNA pre-treatment (Figure 5A, 5B). Similar results were observed following antibody-mediated nucleolin neutralization (Figure 5C, 5D). These data indicated that the inhibitory effect of kallistatin on HUVEC proliferation is largely dependent on nucleolin expression.

We next assessed the effects of the nucleolin-kallistatin interaction on HUVEC migration. To this end, Transwell chambers were set with cells in serum-free media, with the lower compartment containing kallistatin. Results showed that kallistatin induced significant migration inhibition, which was counteracted by nucleolin knockdown (Figure 5E, 5F) or neutralization with a specific antibody (Figure 5G, 5H). These data demonstrated that the inhibitory effects of kallistatin on cell migration also depended on effective nucleolin expression.

### Kallistatin inhibits nucleolin phosphorylation

**Figure 6:**
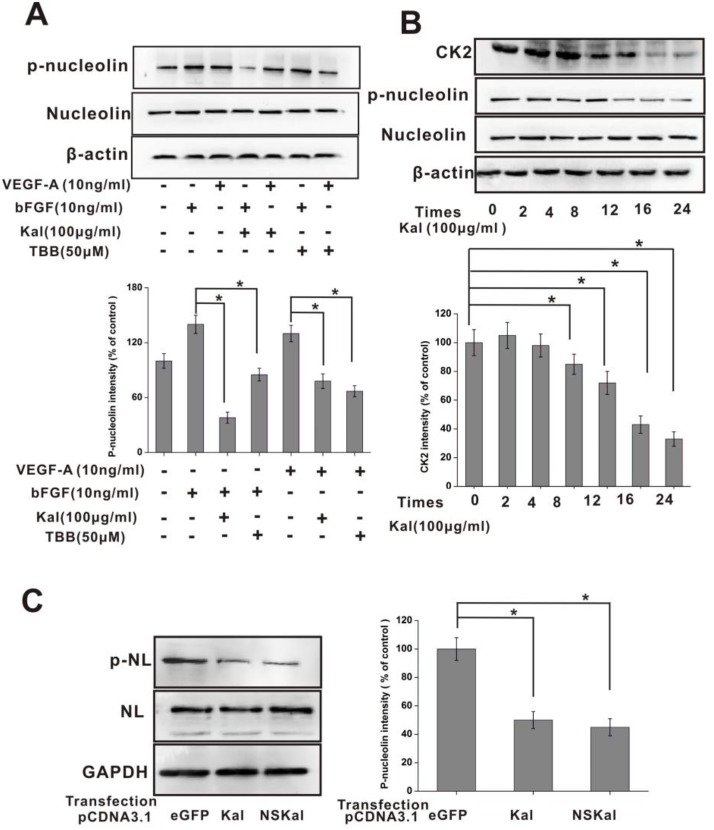
Kallistatin inhibits nucleolin phosphorylation (**A**) IB determination of total nucleolin and phospho-nucleolin expression in HUVECs exposed to growth factors, kallistatin, or a CK2 inhibitor (TBB). β-actin served as a loading control. Data are expressed as mean ± SD from three independent experiments (*N* = 3). ^*^P < 0.05. (**B**) Time-course expression of nucleolin, phospho-nucleolin, and CK2 in HUVECs exposed to kallistatin. Data are expressed as mean ± SD from three independent experiments (*N* = 4). ^*^P < 0.05. (**C**) Western blot analysis of nucleolin phosphorylation in HUVECs transfected with pcDNA3.1-eGFP, pcDNA3.1-Kal, or pcDNA3.1-NSKal. Histogram chart on the right shows corresponding semi-quantification data (mean ± SD from three independent experiments; *N* = 3). ^*^P < 0.05.

After obtaining evidence of positive interaction between kallistatin and nucleolin and the resulting inhibition of HUVEC proliferation and migration, the intracellular phosphorylation of nucleolin was investigated in HUVECs incubated with kallistatin in the presence or absence of growth factors. Western blot results indicated that kallistatin exposure inhibited nucleolin phosphorylation following stimulation by bFGF or VEGF (Figure 6A). Nucleolin plays a prominent role in the nucleolus, promoting ribosomal RNA synthesis upon phosphorylation at N-terminal serine residues following bFGF stimulation. These processes are considered essential for cell proliferation and survival. Past research showed that the phosphorylation of nucleolin was mediated by casein kinase 2 (CK2), as TBB, a specific CK2 inhibitor, abrogated this reaction [29, 30]. Interestingly, our data showed that the expression of CK2 was downregulated when HUVECs were treated with kallistatin (Figure 6B). To further confirm the inhibitory role of kallistatin on nucleolin phosphorylation, a control plasmid (pcDNA3.1-eGFP), or overexpression plasmids encoding kallistatin main isoform (pcDNA3.1-Kal) or a variant unable to localize to the cell surface (pcDNA3.1-NSKal) were introduced into HUVECs. The results demonstrated that both kallistatin fractions significantly attenuated nucleolin phosphorylation (Figure 6C).

### Kallistatin inhibits nucleolin-mediated *in vitro* activation of SRC, FAK, AKT and ERK1/2

**Figure 7:**
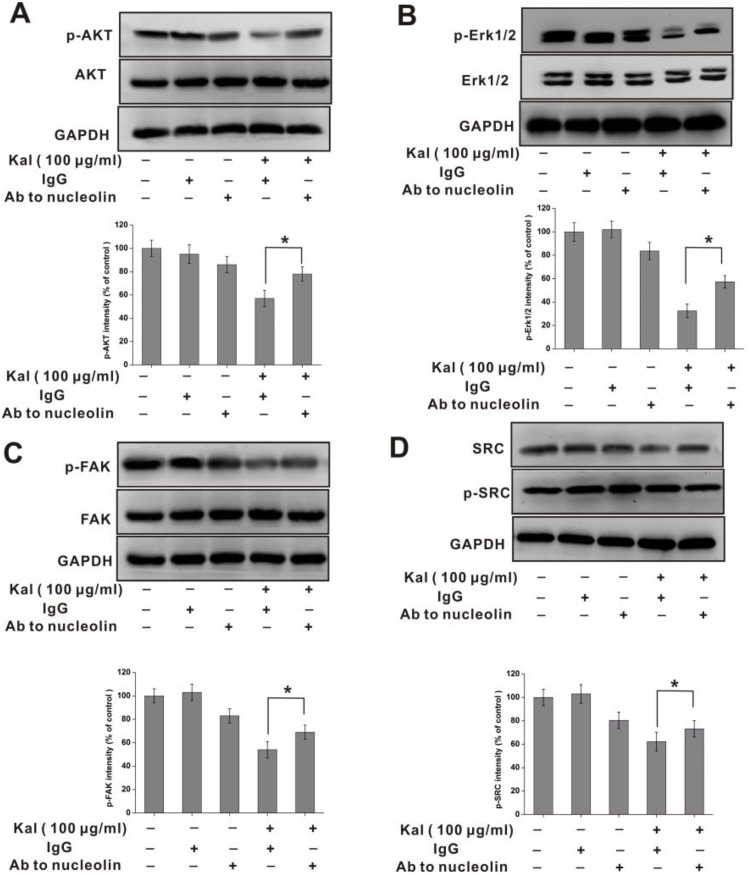
Nucleolin is required for kallistatin-mediated decrease in AKT, ERK1/2, FAK, and SRC phosphorylation Western blot analysis of phosphorylated AKT (**A**), FAK (**B**), ERK1/2 (**C**) and SRC (**D**) in HUVECs incubated with kallistatin and a neutralizing anti-nucleolin antibody. Semi-quantification is shown on the charts below. Data are expressed as mean ± SD from three independent experiments (*N* = 3). ^*^P < 0.05.

Past research showed that members of the SRC, FAK, AKT, and MAPK enzyme families are main components of angiogenesis-related pathways [31–33]. Therefore, we used cultured HUVECs to assess whether kallistatin influences the phosphorylation status of these enzymes. In addition, the involvement of nucleolin in the phosphorylation of the aforementioned proteins was also investigated. Within 12 h following kallistatin exposure, decreased levels of phosphorylated AKT, FAK, SRC, and ERK1/2 were detected (Figure 7). Supporting a role for nucleolin in this phenomenon, these changes were partially reversed in the presence of the anti-nucleolin antibody (Figure 7). Taken together, these data suggested that the anti-angiogenic actions of kallistatin result, at least in part, from nucleolin-mediated suppression of SRC, FAK, AKT and ERK signaling pathways.

### Kallistatin interacts with nucleolin to inhibit tumor growth and neoangiogenesis *in vivo*

**Figure 8:**
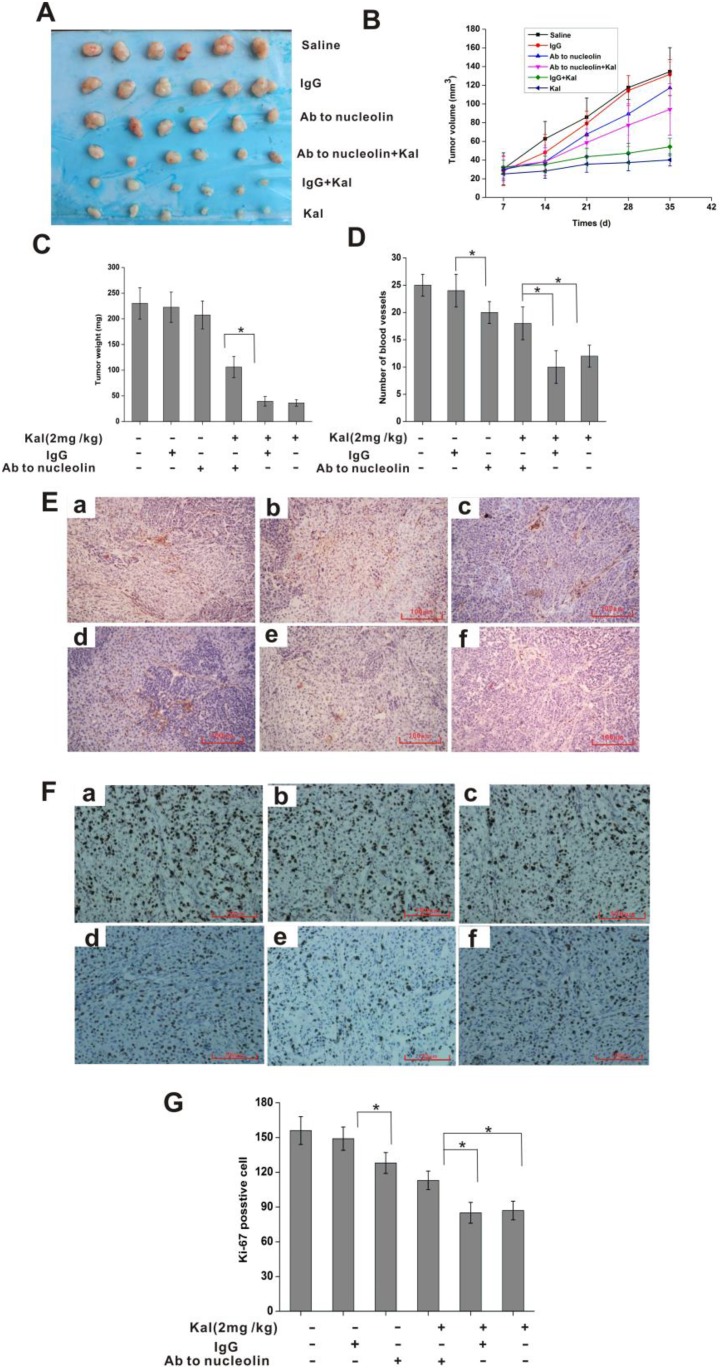
Nucleolin mediates the *in vivo* antitumor and antiangiogenic activities of kallistatin (**A**) Pictures of resected tumors. (**B**) Tumor growth curve. (**C**) Tumor weight measurements. (**D**) Quantification of tumor-associated blood vessel after immunostaining with anti-CD31 as shown in E. Data are expressed as mean ± SD from 6 independent experiments. ^*^P < 0.05. (**E**) Representative images of tumor sections stained with a CD31 antibody. a: saline group; b: IgG group; c: anti-nucleolin antibody group; d: anti-nucleolin antibody plus kallistatin group; e: IgG plus kallistatin group; f: kallistatin group. (**F**) Representative images of tumor sections stained with anti-Ki-67 antibody to detect proliferating cells. a: saline group; b: IgG group; c: anti-nucleolin antibody group; d: anti-nucleolin antibody plus kallistatin group; e: IgG plus kallistatin group; f: kallistatin group. (**G**) Ki-67 proliferation index chart. Data are expressed as mean ± SD from six independent experiments. ^*^P < 0.05.

To assess the inhibitory effects of kallistatin on tumor angiogenesis and growth *in vivo*, and whether nucleolin is required for these actions, we conducted experiments in a HeLa cell xenograft mouse model. To this end, kallistatin, alone or in combination with an anti-nucleolin or a nonimmune, control antibody, was injected i.p. repeatedly following subcutaneous tumor cell inoculation. As shown in Figure 8A–8C, kallistatin administration significantly inhibited tumor growth, while this effect was abrogated by co-injection of anti-nucleolin antibody, but not of nonimmune IgG antibody. Interestingly, immunohistochemical analysis using the hematopoietic progenitor cell marker CD31 showed that tumors from animals co-treated with kallistatin and the anti-nucleolin antibody exhibited a significantly higher blood vessel density than tumor samples from mice treated with kallistatin and nonimmune IgG. (Figure 8D, 8E). Subsequently, the expression of Ki-67, a cell proliferation marker, was investigated in tumor sections by immunohistochemistry. Image analysis revealed significantly higher Ki-67 expression in tumor samples from mice co-treated with kallistatin and anti-nucleolin antibody, compared with those treated with kallistatin and nonimmune IgG antibody (Figure 8F, 8G). These results suggest that kallistatin-mediated inhibition of tumor angiogenesis and tumor growth *in vivo* requires effective surface nucleolin expression on endothelial cells.

## DISCUSSION

The present study demonstrated that nucleolin co-expression at the cell surface of endothelial cells *in vitro* and tumor-associated blood vessels *in vivo* is required for the antiangiogenic and antitumoral actions of kallistatin. Our data indicate that surface membrane expression of nucleolin is required for kallistatin internalization, and the latter in turn inhibits nucleolin phosphorylation, which suggests a complex interplay between these proteins. Nucleolin is a multifunctional phosphoprotein that exerts specific and essential cytosolic and nuclear functions, including organization of nuclear chromatin, rDNA transcription, pre-RNA packaging and transport, and assembly of ribosomes. Moreover, nucleolin also participates in global processes such as cytokinesis, apoptosis, and in the regulation of the transcriptional activity of Myb, KLF2, and the glucocorticoid receptor. Interestingly, nucleolin is selectively expressed on the cell surface of malignant and other rapidly growing cells such as endothelial cells during the angiogenic process. The surface expression of nucleolin and its specific interaction with diverse ligands in various cell types has been the focus of extensive investigations. As a specific marker of angiogenic endothelia, nucleolin has been tested as diagnostic and drug-delivery target molecule. Research showed that N6L, a synthetic pseudopeptide, binds nucleolin on the cell surface and inhibits HUVECs’ adhesion, proliferation and migration *in vitro*. Upon binding nucleolin, these effects result from regulation of AKT, SRC, FAK, and ERK1/2 activities [31]. Endostatin, a heparin-binding 20-kDa peptide with potent antiangiogenic and antitumoral properties, also binds cell surface-expressed nucleolin and undergoes nucleolin-mediated nuclear translocation by virtue of the arginine clusters present on the protein's heparin binding motif. Endostatin trafficking is facilitated by a nuclear localization signal contained on nucleolin's primary amino acid sequence [22, 34–36]. Kallistatin and endostatin are very similar in structure and function. Like endostatin, kallistatin also potently inhibits angiogenesis and possesses heparin-dependent activity. The results presented in the current study demonstrated that the *K*d for the interaction between kallistatin and nucleolin was 2.04 × 10^–8^ M. As with endostatin, this high binding affinity likely results from the arginine residues in the heparin-binding domains of kallistatin.

Tumor angiogenesis has been, for several years, a relevant topic in cancer research. VEGF has been shown to be essential to this process, and many studies have demonstrated that VEGF contributes to the cell surface localization of nucleolin during tumor angiogenesis. This expression pattern appears to depend on cellular growth dynamics and metabolism, which are regulated by a number of kinases involved in cell cycle control, such as CK2 and cyclin-dependent kinase 1 (CDK1). The present results showed that cell-surface levels of nucleolin were reduced following 24 h of serum starvation, while cell surface-bound kallistatin levels decreased in parallel. Moreover, CK2 expression was markedly attenuated on HUVECs after kallistatin exposure. Partial restoration of nucleolin surface membrane localization occurred upon serum restitution and addition of bFGF, suggesting that the cellular distribution of nucleolin *in vitro* is associated with cell growth status.

Our data suggest that the specific mechanisms controlling nucleolin/kallistatin complex formation and internalization, as well as the ensuing signal transduction events may be more complex than initially suggested. It seems conceivable that other hitherto unidentified kallistatin receptors, or formation of co-receptor complexes between nucleolin and other kallistatin-binding proteins such as integrin β3, act in parallel to mediate kallistatin's multiple functions.

## METHODS

### Reagents and antibodies

Recombinant human kallistatin was expressed in *Pichia pastoris* strain GS115 and purified with a series of chromatographic steps, mainly Phenyl Superose and Heparin Sepharose FF chromatography. The antibodies for nucleolin, kallistatin, CK2, GAPDH, MMP2, Integrin β3, LaminB2 were raised in rabbits and were obtained from Cell Signaling Technologies (Boston, MA). Mouse anti-nucleolin antibodies were obtained from Life Technology (Gaithersburg, MD). PVDF membranes and Immobilon ECL were purchased from Millipore Co (Billerica, MA). Protease inhibitor cocktail was obtained from Amresco Inc. (Solon, OH, USA). Dulbecco's modified Eagle's medium (DMEM), DMEM/F12/ Glutamax and fetal bovine serum (FBS) were purchased from Gibco (Carlsbad, CA, USA). Endothelial Growth Medium-2 (EGM-2) and Endothelial Basal Medium-2 were purchased from Lonza Inc (Walkersville, ML). Protein A beads were purchased from Sigma-Aldrich (Steinheim, Germany).

### Animals and cell lines

All animal protocols were in full compliance with the guidelines for animal care and were approved by the Animal Care Committee from the Animal Ethics committee of Huaqiao University. Six–to-eight weeks old male nude BALB/c mice (H-2b) were obtained from the Experimental Center of the Medical Scientific Academy of Fujian. Human umbilical vein endothelial cells (HUVECs, ATCC, CRL-1730) and the cervical cancer cell line HeLa (ATCC, CCL-2) were purchased from American Tissue Type Culture Collection. HUVECs were routinely cultured at 37°C in EGM-2 medium supplemented with 10% FBS. The HeLa cell line was cultured at 37°C in RPMI-1640 medium supplemented with 10% FBS.

### Preparation of membrane proteins

HUVECs were collected with EDTA and washed with cold PBS. The cells were resuspended in 500 μL ice-cold hypotonic buffer (10 mM HEPES, pH 7.9, 0.5 mM dithiothreitol, 0.5 mM phenylmethylsulfonyl fluoride, and one protease inhibitor cocktail). The cells were disrupted with 50 strokes of a tight-fitting Dounce homogenizer (Sango, Beijing, China). The homogenate was monitored under a Nikon phase contrast microscope (Tokyo, Japan) to confirm that no intact cells remained in the samples. The homogenate was centrifuged to remove the nuclei and mitochondria at 8,000 *g* for 10 min. The supernatant was centrifuged at 100,000 *g* for 30 min. The membrane fraction was obtained from the pellet and was dissolved in 200 μL of hypotonic buffer. The membrane proteins were released by treatment with 1% Triton X-100 for 1 h.

### Isolation and identification of kallistatin-binding proteins

Recombinant human kallistatin with a his-tag was expressed in *P. pastoris* strain GS115 and purified by chromatography using Phenyl Superose and Heparin Sepharose FF [9, 37]. A total of 3 mg of his-tagged kallistatin was incubated with Ni-NTA affinity beads for his- pull-down assay. Membrane proteins (10 mg) were incubated with Ni-NTA affinity beads, and these with his-tagged kallistatin for 16 h at 4°C. A control absorption test was performed in parallel with Ni-NTA beads in the absence of kallistatin. Unbound proteins were removed by washing with PBS. Subsequently, the column was washed with different elution buffers and eluted fractions were collected. All protein fractions were run on 12% SDS-PAGE gels, and major bands were digested using sequencing grade porcine-modified tyrosine (Promega, USA). Proteins that showed different abundance between control and sample were identified by peptide mass fingerprint (PMF) using matrix-assisted laser desorption/ionization-time of flight-mass spectrometry (MALDI-TOF). MALDI-TOF data were searched against the Swiss-Prot protein database for protein identification.

### Immunoprecipitation

Immunoprecipitation of the kallistatin complex with its binding protein(s) was conducted as previously described [12]. Whole-cell HUVEC lysates were pretreated with protein A-Sepharose beads for 1 h at 4°C with rotation. Protein A-Sepharose was pelleted by 1,500 g centrifugation to remove potential immunoglobulin contaminants from the original samples. The supernatant was transferred into a flesh microcentrifuge tube followed by incubation with either rabbit immunoglobulin (control) or rabbit anti-kallistatin antibody. The solution was incubated with continuous shaking at 4°C overnight. Protein A beads (Sigma, St. Louis, MO) were subsequently added with continuous shaking for another 2 h. The samples were centrifuged, and the resulting pellets were washed 4 times with PBS and subjected to SDS-PAGE and immunoblotting.

### Immunoblotting

HUVECs were plated at a density of 6 × 10^5^ cells in 6-cm dishes and treated with kallistatin for different time periods. The cells were lysed in whole-cell lysis buffer (50 mM Tris, pH 7.5, 1% NP40, 150 mM NaCl, 10% glycerol, 1 mM EDTA) and supplemented with protease and phosphatase inhibitor cocktail tablets (Amresco). His- pull-down and immunoprecipitation samples were boiled for 10 min prior to the separation by 8–12% SDS/PAGE gels and subsequent transfer was conducted onto PVDF membranes. The membranes were incubated with the designated primary antibodies against kallistatin, nucleolin, phospho-nucleolin, GAPDH, LamB2, CK2, MMP2, and integrin β3. Following washing of unbound primary antibodies, the membranes were incubated with appropriate HRP-conjugated secondary antibodies. Immunoreactive protein bands were visualized by an enhanced chemiluminescence (ECL) detection system and photographed on a Molecular Imager Gel Doc XR system (Bio-Rad). Signal intensity was measured with Image J software to determine relative protein levels.

### Immunofluorescence microscopy

Immunofluorescence microscopy was carried out as described previously [38]. HUVECs were grown on 3-cm dishes overnight and washed three times with PBS. Then cells were fixed with 4% paraformaldehyde in phosphate-buffered saline (PBS), pH 7.4, for 10 min and permeabilized with PBS containing 0.1% Triton. Following 3 washes with PBS, the cells were incubated with anti-kallistatin and/or anti-nucleolin antibodies, followed by Alexa Fluor 488-conjugated anti-rabbit IgG antibody and/or Alexa Fluor 594-conjugated anti-mouse IgG antibody. Cell nuclei were stained with DAPI. Immunofluorescence was documented with a LSM 410 inverted confocal laser-scanning microscope (Carl Zeiss, Oberkochen, Germany) equipped with an Omnichrome argon-krypton laser. Images were obtained with a Zeiss Plan-Apo100X oil immersion objective.

### RNA interference

HUVECs were seeded in 6-well tissue culture plates in the absence of antibiotics. Following 24 h of culture, the cells reached 70–80% confluence and were washed twice with PBS. The double-stranded siRNA sequences were: 5′- GGAACUCACUGGUUUGAAA -3′ for NL, 5′- UGGCUCAGACAUUCGAUCC -3′ for integrin β3, and 5′- CCUACAACUUUG AGA AGGA -3′ for MMP2. Scrambled siRNA, which served as a negative control, was purchased from Life Technologies (Gaithersburg, MD). Double-stranded negative control (NC) siRNA was used concomitantly. Cells were transfected with double-stranded siRNA (15 nmol/L) using Lipofectamine^TM^ 2000 (5 μL/well) in Opti-MEM medium, following the manufacturer's instructions [10]. The cells were incubated for an additional 24 h prior to further experiments.

### Cell viability

Cell viability was monitored using the MTT (3-(4, 5-dimethylthiazol-2-yl)-2, 5-diphenyl- tetrazolium bromide) assay. HUVECs (3 × 10^3^) were incubated in 96-well plates with or without kallistatin in EGM-2 medium supplemented with 10% fetal bovine serum, penicillin (100 U/ml), and streptomycin (100 μg/ ml) in a humidified atmosphere of 5% CO_2_ at 37°C. Following 48 h of incubation, MTT was added (10 μg/well) and the plate was incubated at 37°C for 3 h. The supernatants were discarded and DMSO was added (200 μl/well) and mixed for 10 min vigorously. MTT crystals’ absorbance was quantified using an automatic plate reader (Molecular Devices) at a 490 nm test wavelength and a 630 nm reference wavelength.

### Cell proliferation assay

The proliferation of HUVECs was assessed using the Click-iTEdU Alexa Fluor 594 Imaging Kit according to the instructions provided by the manufacturer. Briefly, cells were pre-treated with kallistatin or PBS for 48 h, and incubated with 10 μM EdU for 4 h at 37 °C. The cells were subsequently fixed with 4% formaldehyde for 15 min, and treated with 0.5% Triton X-100 for 20 min at room temperature for permeabilization. Following three washes with PBS, the cells were incubated with the Apollo reaction cocktail for 30 min. DNA was stained with 10 μg/ml of Hoechst 33342 stain for 20 min and visualized by fluorescence microscopy. At least 6 random fields per well were captured at 100× magnification, and Image- Pro Plus 6.0 software (IPP 6.0) was used to calculate the percentage of EdU-positive cells (identified by Apollo^®^ 594 fluorescence) within the total cell population (identified by Hoechst 33342 nuclei staining). For certain experiments, the cells were pre-treated with siRNA and/or anti-nucleolin antibody for 24 h prior to kallistatin treatment.

### Cell migration assay

Migration assays were carried out using Transwells (Costar, NY, USA; pore size, 8 μm) in 24-well dishes. HUVECs were harvested and resuspended at a concentration of 10^4^ cells/0.2 ml in serum-free medium. The bottom chamber was filled with 0.3 ml of the corresponding medium, containing 12.5, 50, or 200 μg/ml of kallistatin. The upper chamber was loaded with 0.1 ml of medium containing the cells and incubated for 12 h at 37°C in the presence of 5% CO_2_. The cells were fixed in 20% methanol for 15 min and stained with 0.1% crystal violet in PBS (v/v) for 15 min. The cells on the upper side of the filters were removed with cotton-tipped swabs, and the filters were washed with PBS. The cells on the underside of the filters were examined and counted under a microscope. Each experiment was carried out in triplicate, and repeated independently at least three times. IPP 6.0 software was used to quantify migrated cells.

### Tumor growth assay

HeLa cells (1 × 10^6^ in 50 μL) were inoculated subcutaneously in 6–8 weeks old nude mice. Intraperitoneal injections were applied 24 h later. The injections contained either saline, nonimmune rabbit IgG antibody (2 mg/kg), rabbit anti-nucleolin antibody (2 mg/kg), kallistatin (2 mg/kg), rabbit anti-nucleolin antibody (2 mg/kg) plus kallistatin (2 mg/kg), or nonimmune rabbit IgG antibody (2 mg/kg) plus kallistatin (2 mg/kg). Animal weights were monitored, and tumor dimensions were measured (starting on day 1) in two perpendicular dimensions every 3 days using calipers. Tumor volume was calculated according to the following formula: V = (a^2^ × b)/2, where ‘a’ is tumor the width (smaller diameter) and ‘b’ is tumor length (larger diameter). Following 35 days of tumor growth, the mice were sacrificed and the tumors were resected, weighed, fixed, and prepared for immunohistochemical analysis. Tumor blood vessels were labeled with an anti-CD31 antibody, and the average number of blood vessels observed in 5 random high-power (×100) fields per section was determined by optical microscopy.

### Statistical analysis

Data are presented as mean ± SD. Comparisons among groups were conducted by one-way analysis of variance (ANOVA), followed by Scheffe's test. *P* < 0.05 was considered statistically significant.

## Abbreviations

NL: nucleolin
CK2: casein kinase 2
CDK1: cyclin-dependent kinase 1
AKT: protein kinase B
FAK: focal adhesion kinase
eNOS: endothelial nitric oxide synthase
LRP6: low-density lipoprotein receptor-related protein 6
KLF-2: Krüppel-like factor 2
Erk: extracellular signal regulated kinase
EdU: 5-ethynyl-2-deoxyuridine
PI3K: phosphoinositide 3-kinase
PBS: phosphate-buffered saline
eNOS: endothelial nitric oxide synthase
KLF-4: Krüppel-like factor 4
HSPGs: Heparan sulfate proteoglycans.
